# Attention-deficit/hyperactivity disorder and impairment in executive functions: a barrier to weight loss in individuals with obesity?

**DOI:** 10.1186/1471-244X-13-286

**Published:** 2013-11-07

**Authors:** Samuele Cortese, Erika Comencini, Brenda Vincenzi, Mario Speranza, Marco Angriman

**Affiliations:** 1 Child Neuropsychiatry Unit, G. B. Rossi Hospital, Department of Life Science and Reproduction, Verona University, Verona, Italy; 2 Phyllis Green and Randolph Cowen Institute for Pediatric Neuroscience, Child Study Center of the NYU Langone Medical Center, New York, NY, USA; 3 Massachusetts General Hospital, Schizophrenia Clinical and Research Program, Boston, MA, USA; 4 EA4047, University of Versailles Saint-Quentin-en-Yvelines, Versailles, France; 5 Child and Adolescent Psychiatry, Versailles General Hospital, Le Chesnay, France; 6 Child Neurology and Neurorehabilitation Unit, Department of Pediatrics, Central Hospital of Bolzano, Bolzano, Italy; 7 Unità Autonoma di Neuropsichiatria Infantile, Ospedale G.B . Rossi, P.le L.A. Scuro, 12, Verona 37134, Italy

**Keywords:** ADHD, Executive functions, Obesity, Treatment resistance

## Abstract

**Background:**

An increasing body of research points to a significant association of obesity to Attention-Deficit/Hyperactivity Disorder (ADHD) and deficits in executive functions. There is also preliminary evidence suggesting that children with ADHD may be at risk of obesity in adulthood.

**Discussion:**

In this article, we discuss the evidence showing that ADHD and/or deficits in executive functions are a barrier to a successful weight control in individuals enrolled in weight loss programs. Impairing symptoms of ADHD or deficits in executive functions may foster dysregulated eating behaviors, such as binge eating, emotionally-induced eating or eating in the absence of hunger, which, in turn, may contribute to unsuccessful weight loss. ADHD-related behaviors or neurocognitive impairment may also hamper a regular and structured physical activity. There is initial research showing that treatment of comorbid ADHD and executive functions training significantly improve the outcome of obesity in individuals with comorbid ADHD or impairment in executive functions.

**Summary:**

Preliminary evidence suggests that comorbid ADHD and deficits in executive functions are a barrier to a successful weight loss in individuals involved in obesity treatment programs. If further methodologically sound evidence confirms this relationship, screening and effectively managing comorbid ADHD and/or executive functions deficits in individuals with obesity might have the potential to reduce not only the burden of ADHD but also the obesity epidemics.

## Background

Attention-Deficit/Hyperactivity Disorder (ADHD) is defined by persistent, age inappropriate and impairing levels of inattention and/or hyperactivity-impulsivity [1]. The Diagnostic and Statistical Manual of Mental Disorders-4^th^ edition, Text Revision, IV-TR [1] defines four types of ADHD: “predominantly inattentive”, “predominantly hyperactive-impulsive”, “combined”, and “not otherwise specified”. Although outside the scope of this article, since the final text is not yet available, we note that the core structure of the diagnostic criteria is largely unchanged in the forthcoming fifth edition of the diagnostic manual.

ADHD is one of the most frequent childhood-onset psychiatric conditions, with an estimated worldwide-pooled prevalence exceeding 5% in school-age children [2]. Impairing symptoms of ADHD persist into adulthood in up to 65% of childhood-onset cases [3] and the pooled prevalence of ADHD in adults has been estimated at ~2.5% [4].

Executive functions are defined as a set of neurocognitive skills that are necessary to plan, monitor and execute a sequence of goal-directed complex actions and include inhibition, working memory, planning, and sustained attention [5]. Besides the behavioural core symptoms of inattention, hyperactivity, and impulsivity, deficits in executive functions are commonly, although not universally, associated with ADHD [6]. Indeed, executive dysfunction is not required for the diagnosis of ADHD, which is defined at the behavioral, rather than neuropsychological, level. Additionally, ADHD is usually comorbid with other neurodevelopmental and/or psychiatric conditions, such as learning disorders, oppositional defiant/conduct disorder, mood and anxiety disorders, substance use disorders, and sleep disturbances [7,8].

Currently, the mainstay of treatment, at least for severe cases, is pharmacologic, with psychostimulant medications (methylphenidate and amphetamines) as the first line, and non-stimulants as secondary option [6,9]. Non-pharmacological treatments, such as behavioural therapies, diet regimens, cognitive training, and neurofeedback, are also available. Although the empirical evidence for their efficacy for ADHD core symptoms is so far weak [10], such treatments may effectively address related behavioural or neuropsychological dysfunctions.

Because of its core symptoms as well as associated disorders/conditions, ADHD entails an enormous burden on society in terms of psychological dysfunction, adverse vocational outcomes, stress on families, and societal financial costs. The U.S. annual incremental costs of ADHD have been recently estimated at $143-$266 billion [11] and high costs have been reported in other countries as well (e.g., [12]).

Whereas the comorbidity between ADHD and psychiatric disorders has been extensively explored [7], the association with general medical conditions has received much less attention. However, among medical disorders, there is increasing evidence pointing to a significant association between overweight/obesity and ADHD in children [13,14] as well as in adults [15-17]. In particular, as detailed in a previous systematic review [18] and outlined in Table 1, all currently available studies show significantly higher rates of ADHD in individuals with obesity treated in specialized centres compared to normal weight controls or population rates of ADHD. (Studies listed in Table 1 were retrieved searching Pubmed, Ovid, EMBASE, and Web of Knowledge, from their inception to March 15^th^, 2013, using the following keywords, in multiple combination combination: *obesity, BMI, weight, body mass, ADHD, Attention-Deficit/Hyperactivity Disorder, Attention Deficit Disorder, Hyperkinetic Disorder*; details of the search strategy and syntax, adapted for each database, as well as of the specific results from each database search, are available from the corresponding author). Given the cross-sectional design of such studies, they cannot allow to infer the causal relationship between obesity and ADHD. Theoretically, it is possible that: 1) ADHD contributes to weight gain; 2) Obesity early in life fosters symptoms of ADHD; 3) Both conditions are the expression of underlying neurobiological and psychopathological dysfunctions. Recent studies have shaded light on the causal relationship between ADHD and obesity, supporting in part the notion that ADHD in childhood may contribute to weight gain later on in life. Cortese et al. [19] assessed body mass index (BMI) and obesity rates in a sample of 111 U.S. adults with childhood problems consistent with DSM-IV(-TR) ADHD, combined type, followed up for 33 years, and matched comparisons (N = 111) without childhood ADHD. They found that BMI and obesity rates were significantly higher in individuals with childhood ADHD *vs.* non ADHD comparisons (41.4% vs. 21.6%, respectively), even after controlling for possible confounders such as socio-economic status (SES) and comorbid psychiatric disorders associated with obesity, i.e., mood, anxiety, and substance use disorders. However, anthropometric data were not collected in childhood, which prevented the authors from determining whether the association between childhood ADHD and weight status at follow-up in adulthood was attributable to weight status in childhood or whether it developed later. Using a dimensional approach (i.e., considering the intensity of each ADHD symptom) rather than a categorical approach based on the DSM-IV-TR nosography, Fuemmelar et al. [16] found a significant linear relationship between the number of retrospectively reported symptoms of inattention or hyperactivity/impulsivity in childhood and adulthood BMI in a population based sample of 15,197 individuals (National Longitudinal Study of Adolescent Health). Extending such evidence, Cortese et al. [20] analyzed a sample of 34,653 U.S. adults from the National Epidemiologic Survey on Alcohol and Related Conditions and found a significant association between the number of symptoms of inattention, hyperactivity, or impulsivity (retrospectively reported) in childhood and obesity in adulthood. However, after controlling for SES and an extensive set of psychiatric disorders, the association held only in women, thus calling for future studies taking into account possible gender differences. The retrospective report of ADHD symptoms is a limitation of this study.

**Table 1:** Studies assessing the rates of Attention-Deficit/Hyperactivity Disorder (ADHD) in clinical samples of treatment-seeking individuals with obesity

**First author (year) [ref]**	**Sample characteristics**	**Key findings**
**Altfas (2004) **[21]	215 patients with obesity treated in a specialized obesity clinic (Males: 22; mean age: 43.4 ± 10.9 years)	Prevalence of ADHD in the whole sample: 27.4%. Prevalence of ADHD in individuals with BMI ≥ 40 kg/m^2^: 42.6%. Mean BMI loss among patients with ADHD: 2.6 BMI (kg/m^2^) *vs.* 4.0 for non-ADHD (p < 0.002)
**Erermis (2004) **[22]	30 adolescents with obesity (Males: 14; mean age: 13.8 ± 1.2 years) seeking treatment in a paediatric endocrinology outpatient clinic	Prevalence of ADHD: 13.3%
**Agranat-Meged (2005) **[23]	26 adolescents in a tertiary referral centre for obesity (Males: 13; mean age: 13.04 ± 2.8 years ), all with morbid obesity (BMI > 95 percentile)	57.7% of the subjects presented with ADHD diagnosed with semi-structured interviews
**Fleming (2005) **[24]	75 women with severe obesity (BMI ≥ 35 kg/m^2^) (mean age: 40.4 ± 7.25 years) referred for non surgical treatment of obesity	26.7% of women reported impairing symptoms of ADHD in both childhood and adulthood
**Alfonsson (2012) **[25]	187 individuals (Males: 50; mean age: 44.28 ± 6.02 years) with obesity, candidate for bariatric surgery	10% of the subjects presented with ADHD. ADHD symptoms significantly correlated with anxiety, depression, and disordered eating (“lack of control over eating”, “eating alone because embarrassed”, “eating until feeling uncomfortable”, and “feeling guilty after overeating”)
**Gruss (2012) **[26]	116 patients (Males: 31; mean age: 44.28 ± 6.02 years) candidate for bariatric surgery	12% of the patients screened positive for ADHD. Rates of Binge Eating disorder did not differ between patients with and without ADHD
**Nazar (2012) **[27]	150 women (mean age: 38.9 ± years)	Prevalence of ADHD: 28.3%. ADHD was significantly correlated with more severe binge eating, bulimic behaviors, and depressive symptoms severity

With regards to executive dysfunctions, there is an emerging literature indicating their possible association with overweigh/obesity. In a recent systematic review [28] including 31 papers limited to children and adolescents, Reinert and colleagues concluded that inhibitory control, assessed with several neuropsychological tests, was the most consistently impaired executive function across studies. About 77% of the retained studies in children and 73% of the papers relative to adolescents with obesity reported a significant impairment in this executive function. Additionally, scores on neuropsychological tests assessing inhibitory control were significantly lower (p < 0.01) in children with obesity than in normal weight comparisons, when pooling data across studies. Another recent systematic review [29] considering individuals across the lifespan and using a different approach in the selection of the papers, showed that decision making, planning and problem solving were the most compromised domains, although the authors note the high heterogeneity across studies in the methodology and in the selection of the neuropsychological tests.

Summarizing, there is increasing evidence that both ADHD, at least considering samples of treatment-seeking individuals, and deficits in executive functions, even in the absence of a formal diagnosis of ADHD, may be associated with obesity. Additionally, there is preliminary evidence that ADHD may causally contribute to obesity/overweight. However, an important aspect to note is that the impact of ADHD or executive dysfunction on obesity outcome is still underexplored. In the next section, we discuss the preliminary evidence showing that ADHD or executive dysfunction may represent an important barrier to successful weight loss in patients with obesity during weight loss programs. We also point out the clinical implications of these findings, as well as possible future research directions in this emerging area of investigation.

## Discussion

Several possible dysfunctional behavioural pathways associated with either ADHD (as a categorical diagnosis) or related neuropsychological deficits in executive functions lead to hypothesize that impairing symptoms of impulsivity, inattention or hyperactivity (the behavioural core symptoms of ADHD) and/or related neurocognitive impairment may be a barrier to successful weight loss during treatment interventions for individuals with obesity.

First, it is possible that impulsivity and deficient neurocognitive inhibitory control foster impulsive and dysregulated eating behaviors, which, in turn, would hamper the success of dietetic regimen. These abnormal eating behaviors include binge eating, “external eating” (i.e., eating in response to food-related stimuli, regardless of the internal state of hunger or satiety) and “emotionally-induced eating” (i.e., excessive eating as a response to emotional states), all of which have been related to obesity and overweight [30,31].

Second, another dysfunction related to impulsivity and deficits in inhibitory control, namely altered reward sensitivity, may also contribute to dysregulated eating behaviors. Indeed, a subset of individuals with ADHD present with a preference for small immediate over larger delayed rewards [29,32]. This could therefore hamper dietetic efforts when considering eating-related rewards deriving from appetizing foods.

Third, it has been noted that attention and related executive functions such as planning and organizational skills are important for a successful adhesion to dietetic regimen and regular physical exercise [18], both of which underpin effective and sustained weight control.

The previous hypotheses of a correlation between ADHD symptoms and/or executive function deficits and abnormal eating start being supported by empirical evidence. With regards to ADHD symptoms, Cortese et al. [33] found a significant correlation between inattentive and impulsive ADHD symptoms and binge eating behaviors, even after controlling for comorbid depression and anxiety, in a study of 99 consecutively referred severe obese adolescents (12-17 years). By means of structural equation modelling, Davis et al. [30] found a significant correlation between ADHD symptoms and abnormal eating behaviors (including binge eating and emotionally-induced eating) in a sample of healthy adult women (25-46 years). Using the same modelling, Strimas et al. [34] confirmed these results also in a sample of 145 non-clinical adult males.

There is also evidence that deficit in executive dysfunctions are related to abnormal eating behaviors, although causal relationship has not been tested. For example, in a study of 55 women reporting weekly binge eating in the absence of regular compensatory behaviors, Kelly et al. [35] found a significant correlation between frequency of binge eating behaviors and deficit in executive functions such as flexibility in thinking and shifting attention. By means of path analyses, Dempsey et al. [36] confirmed a significant correlation between deficit in executive functions and overeating behaviors in a sample of 135 individuals from the community. This evidence has been extended to young children. Pieper and Laugero [37] recently reported a significant correlation between executive function deficits, measured by means of child-completed tasks and parental as well as teacher reports, and eating in the absence of hunger in a sample of 29 preschool children (3-6 years).

While the correlation of impulsivity and inattention domains to abnormal eating behaviors associated with obesity may be intuitive, one could think that the hyperactive component of ADHD is not involved at all and, actually, it may favour weight loss rather than weight gain. However, it is well known that the motor hyperactivity of ADHD is not constant. Actigraphic measures have shown that motor hyperactivity is modulated by situational variables and may be indistinguishable from normal when there is sufficient stimulation. For example, no significant differences in hyperactivity levels between children with ADHD and healthy comparisons have been detected while watching television, whereas children with ADHD show significantly more hyperactivity during classes at school [38]; it is important to note that children with ADHD have also been shown to watch more television than non ADHD children. Interestingly, psychostimulant medications induce an increase, rather than a decrease, and a normalization of motor activity during physical education, where movement is appropriate and expected [38]. It is also possible that excessive motor activity in the morning during breakfast hampers a correct consumption of breakfast; in turn, skipping breakfast has been shown as a risk factor for weight gain and obesity [39]. Additionally, restlessness during lunch and dinner may decrease regular food consumption during these structured moments, with inappropriate and excessive compensatory calories intake outside meals. Therefore, we hypothesize that the balance between the tendency to overeat in an irregular way following irregular breakfast and meals, from one side, and the inconstant energy expenditure associated with motor hyperactivity in ADHD, on the other side, may explain why ADHD hyperactivity contributes to increase the risk of obesity.

So, if ADHD or related executive functions deficits foster abnormal eating behaviors contributing to obesity, is there evidence supporting that ADHD and executive dysfunction also represent a barrier to effective and lasting weight loss in individuals with obesity? This starts being reported in the literature, although further and more methodologically sound evidence is needed.

In an observational study of 215 adults with obesity in a specialized clinics, Altfas [21] was the first to note that those without comorbid ADHD achieved nearly twice the BMI loss compared to patients with comorbid ADHD, despite the fact that the latter engaged in more visits, thus suggesting a pattern of “taking more time to accomplish less” associated with ADHD. Afterwards, in a study of adults involved in a behavioral weight loss program, Pagoto et al. [40] confirmed that participants with ADHD reported more previous weight loss attempts and lost less weight than those who did not screen positive for ADHD. Another recent study showed that patients presenting for bariatric surgery (BS) with comorbid ADHD had significantly more difficulties in following visits after BS than those without comorbid ADHD [41].

Indirect support to the hypothesis that executive function deficit is a barrier to effective weight control is also provided by a longitudinal prospective study by Speranza et al. [42] who found that alexithymia was a significant predictor of treatment outcome at 3-year follow-up in a sample of youths with eating disorder. Indeed, alexithymia is related to executive function deficits, as summarized in [43].

Given this preliminary literature, a crucial question is whether treatment of ADHD and/or improvement in executive functions are also effective in decreasing/preventing obesity in children with both conditions. There is initial evidence indicating that the answer may be affirmative.

In a study [44] of 242 individuals with a lengthy history of weight loss failure consecutively referred for refractory obesity, 78 patients (32.2%) screened positive for ADHD. Of these, 65 started pharmacotherapy for ADHD with psychostimulants, in addition to standard management for weight loss, and were followed up for an average of 466 days. Those who refused pharmacological treatment or who did not tolerate it for adverse events (n = 13) were also followed up, serving as comparisons, and received standard care for weight loss management. At follow-up, individuals who received treatment lost 12.36% of their initial weight, whereas comparisons gained an average of 2.78% (p < 0.001). A putative confounder in interpreting these results is the possible anorexigenic effect that may be associated with psychostimulant treatment. However, appetite reduction was evident in the first 4–6 weeks of treatment, but then it diminished and vanished in most of the subjects within 2 months. Therefore, the authors of the study concluded that it is unlikely that the anorexigenic effect of psychostimulants contributed to the weight loss at follow-up, after more than one year from the start of the treatment. A limitation of this study is its design: although the study was controlled, it was not randomized. Indeed, since the pharmacological treatment for ADHD is effective and is recommended in several guidelines, [6,9], for ethical reason it was not possible to randomize participants either to pharmacological treatment or placebo. As such, this study cannot provide high-level evidence. Although a randomized study testing the effects on weight of psychostimulants for ADHD would be challenging, interestingly, there is preliminary evidence from a randomized trial [45] suggesting that the training of executive functions is highly effective to improve the outcome of obesity. In this trial, Verbeken and coworkers assessed the effects of executive functions training with video games aimed at improving inhibitory control and working memory. They randomized 44 children (8-14 years) who were in the final part of a 10- month inpatients treatment program in an obesity centre to either 6-week executive functions training or to standard care for weight control. At 8 weeks after the training, children in the training group showed significantly better weight loss maintenance than those in the standard care group.

### Clinical implications

If further methodologically sound studies confirm that ADHD and/or related executive functions deficits are a barrier to effective weight loss, it would be worthy for the clinicians and professionals involved in the management of obesity to screen for ADHD and impairment in executive functions. We note that professionals involved in the treatment of obesity usually do not have an appropriate knowledge of ADHD and related disorders. A systematic screening and appropriate treatment of ADHD and/or executive functions deficits might not only reduce the burden of ADHD, but also improve the outcome of patients with a past history of weight loss failure. This is particularly relevant in terms of decreasing the stigma associated with obesity. Unfortunately, a common belief manifested not only by the lay public but also by some professionals is that individuals with obesity may fail to succeed at weight-loss programs due to their “laziness” [46]. Inattention and related impaired executive functions, as well as impulsivity that hamper the appropriate adherence to a regular diet regimen, might be mistakenly attributed to laziness and “character problems”. Therefore, awareness that unsuccessful weight loss may be due, at least in part, to neurocognitive impairment could contribute to decrease the stigma associated with obesity.

### Future research in the field

We believe that the relationship between ADHD/executive functions and obesity, as well as the impact of neurocognitive impairment on weight loss management, is still in a developing phase. While the cross-sectional relationship between ADHD and obesity starts being well characterized from a clinical descriptive standpoint, further longitudinal studies are needed to better assess the causal relationship. Studies aimed at elucidating common neurobiological and genetics underpinnings are still in their infancy (e.g., [47]) and need further attention. Perhaps even more important in terms of implementation science would be to assess, by means of rigorous randomized controlled trials, the effects of ADHD treatment or executive functions training on weight outcome in individuals enrolled in weight loss programs. In particular, it would be highly relevant to establish if early management of ADHD in young children leads to further better obesity outcome later on. However, given the challenges, from an ethical standpoint, of conducting long-term randomized controlled trials where participants are assigned either to an effective treatment for ADHD or to placebo, longitudinal studies comparing the weight outcome of obese children treated with ADHD medications vs those who opt for non pharmacological approaches or no treatment, matched for baseline BMI and socio economic status, could provide useful data. This design will likely require multi-center recruitment. Research in such area is worthy and could contribute to decrease the worldwide obesity epidemics.

## Summary

Preliminary evidence suggests that comorbid ADHD and deficits in executive functions are a barrier to a successful weight loss in individuals involved in obesity treatment programs. If further methodologically sound evidence confirms this relationship, screening and effectively managing comorbid ADHD and/or executive functions deficits in individuals with obesity might have the potential to reduce not only the burden of ADHD but also the obesity epidemics.

## Abbreviations

ADHD: Attention-Deficit/Hyperactivity Disorder; BMI: Body mass index; SES: Socioeconomic status.

## Competing interests

Dr. Cortese has received financial support to attend medical meetings from Eli Lilly & Company (2008) and Shire Pharmaceuticals (2009-2010), and has been co-investigator in studies sponsored by GlaxoSmithKline (2007), Eli Lilly & Company (2008), and Genopharm (2009). He has served as scientific consultant for Shire Pharmaceuticals (2009-2010). Drs. Comencini, Vincenzi, Speranza, and Angriman declare no competing conflict of interest.

## Authors’ contributions

Dr. Cortese conceived and drafter the manuscript and approved the final version to be submitted; Drs. Comencini, Vincenzi, Speranza, and Angriman critically revised the first draft, contributed to the literature search, and approved the final version to be submitted. All authors read and approved the final manuscript.

## Authors’ information

Dr. Cortese is a post doctoral fellow at the Institute for Pediatric Neuroscience, New York University, New York, NY, USA and at the Child Neuropsychiatry Unit, Verona University, Italy. His research interests focus on ADHD, in particular on the neurobiology and on the evidence-based treatment of ADHD. Dr. Comencini is a resident in child psychiatry at the Child Neuropsychiatry Unit, Verona University, Italy. Her research focuses on the psychopathology of children with obesity. Dr. Vincenzi is a research fellow at the Massachusetts General Hospital, Schizophrenia Clinical and Research Program, Boston, MA, USA. Her research focuses on eating disorders and schizophrenia. Dr. Speranza is a consultant at the Child and Adolescent Psychiatry, Versailles General Hospital. Le Chesnay, France and a researcher at the University of Versailles Saint-Quentin-en-Yvelines, Versailles, France. His research focuses on eating disorders, ADHD, and personality disorders. Dr. Angriman is a consultant at the Child Neurology and Neurorehabilitation Unit, Department of Pediatrics, Central Hospital of Bolzano, Italy. His research focuses on ADHD, obesity, and sleep disorders.

## Pre-publication history

The pre-publication history for this paper can be accessed here:

http://www.biomedcentral.com/1471-244X/13/286/prepub
